# Phenotypic and Genotypic Properties of *Vibrio cholerae* non-O1, non-O139 Isolates Recovered from Domestic Ducks in Germany

**DOI:** 10.3390/microorganisms8081104

**Published:** 2020-07-23

**Authors:** Nicola Hirsch, Eva Kappe, Armin Gangl, Keike Schwartz, Anne Mayer-Scholl, Jens Andre Hammerl, Eckhard Strauch

**Affiliations:** 1 Tiergesundheitsdienst Bayern, Bavarian Animal Health Service, 85586 Poing, Germany; Nicola.Hirsch@tgd-bayern.de (N.H.); Eva.Kappe@tgd-bayern.de (E.K.); Armin.Gangl@tgd-bayern.de (A.G.); 2 Department of Biological Safety, German Federal Institute for Risk Assessment, Max-Dohrn-Str. 8-10, D-10589 Berlin, Germany; keike.schwartz@bfr.bund.de (K.S.); Anne.Mayer-Scholl@bfr.bund.de (A.M.-S.); jens-andre.hammerl@bfr.bund.de (J.A.H.)

**Keywords:** diseased birds, virulence factors, phylogenetic analysis, antimicrobial resistance

## Abstract

*Vibrio cholerae* non-O1, non-O139 bacteria are natural inhabitants of aquatic ecosystems and have been sporadically associated with human infections. They mostly lack the two major virulence factors of toxigenic *V. cholerae* serogroups O1 and O139 strains, which are the causative agent of cholera. Non-O1, non-O139 strains are found in water bodies, sediments, and in association with other aquatic organisms. Occurrence of these bacteria in fecal specimens of waterfowl were reported, and migratory birds likely contribute to the long-distance transfer of strains. We investigated four *V. cholerae* non-O1, non-O139 isolates for phenotypic traits and by whole genome sequencing (WGS). The isolates were recovered from organs of domestic ducks with serious disease symptoms. WGS data revealed only a distant genetic relationship between all isolates. The isolates harbored a number of virulence factors found in most *V. cholerae* strains. Specific virulence factors of non-O1, non-O139 strains, such as the type III secretion system (TTSS) or cholix toxin, were observed. An interesting observation is that all isolates possess multifunctional autoprocessing repeats-in-toxin toxins (MARTX) closely related to the MARTX of toxigenic El Tor O1 strains. Different primary sequences of the abundant OmpU proteins could indicate a significant role of this virulence factor. Phenotypic characteristics such as hemolysis and antimicrobial resistance (AMR) were studied. Three isolates showed susceptibility to a number of tested antimicrobials, and one strain possessed AMR genes located in an integron. Knowledge of the environmental occurrence of *V. cholerae* non-O1, non-O139 in Germany is limited. The source of the infection of the ducks is currently unknown. In the context of the ‘One Health’ concept, it is desirable to study the ecology of *V. cholerae* non-O1, non-O139, as it cannot be excluded that the isolates possess zoonotic potential and could cause infections in humans.

## 1. Introduction

*Vibrio cholerae* are gram-negative bacteria found in aquatic ecosystems worldwide. Strains belonging to the serogroups O1 and O139 are the causative agent of cholera, which is a dreaded disease in developing countries and is responsible for thousands of illnesses and human deaths every year. The genes for the cholera toxin and the toxin-coregulated pilus are regarded as the major virulence factors of the toxigenic strains [1]. However, numerous *V. cholerae* strains exist, which do not possess these two virulence factors. The nontoxigenic strains belong to other serogroups and are commonly designated as *V. cholerae* non-O1, non-O139. Some of these strains sporadically cause gastrointestinal infections or wound infections in humans [2].

Diseases of animals associated with *V. cholerae* non-O1, non-O139 are rarely reported. Though a number of *Vibrio* species are well-known pathogens for fish, a role of *V. cholerae* non-O1, non-O139 as a possible fish pathogen is reported less frequently. A recent review [3] gives an overview of the isolation of *V. cholerae* non-O1, non-O139 from diseased fish. Although, the authors suspected that, in some cases, the diseases might have been caused by other bacterial species or viruses. The increase in aquaculture production has led to a rise in infections by *V. cholerae* non-O1, non-O139 in farmed shrimps and infections in giant freshwater prawns and whiteleg shrimps being reported [4,5].

As *V. cholerae* is found worldwide in aquatic ecosystems, the occurrence of strains of this species in waterfowl is not surprising. Several years ago, *V. cholerae* were identified from cloacal swabs and feces taken from different species of aquatic birds [6,7]. In a recent study, several Vibrio species, including *V. cholerae,* were recovered from debilitated wrecked marine birds in Brazil [8]. Pretzer et al. (2017) [9] reported the occurrence of highly diverse *V. cholerae* non-O1, non-O139 populations in an Austrian lake (Neusiedler See) and migrating birds were hypothesized to play a key role in the transfer of the strains over long distances.

Diseases of water birds caused by *V. cholerae* non-O1, non-O139 have rarely been reported. *V. cholerae* NAG (non-agglutinable with antisera against O1 strains) were isolated from ducklings from Danish farms suffering from conjunctivitis. Isolates were found in the conjunctival fluid of puffins and were also present in the gut of the birds. Investigations of the environment made it likely that the bacteria were taken up from brackish water in their surroundings [10]. A subsequent investigation revealed that ducks inside the farm buildings had been free of *V. cholerae* and only after the birds were released into the open fields *V. cholerae* were detected in the cavum nasi and pharynx of the birds [11]. While in the first study some *V. cholerae* were isolated together with other bacteria from conjunctiva of diseased ducks, in the second study the animals were apparently healthy. A more likely association of a *V. cholerae* non-O1 infection in birds was reported [12] when a strain was isolated from the liver of a deceased goose. The goose was from a flock with six ill birds among 17 animals. Other poultry on the farm (chicken, mallards, and guinea fowls) were not affected. Another study reported two *V. cholerae* isolates among a number of other gram-negative bacteria that had been isolated from several cases of septicemia and airsacculitis in ducks [13]. As the study aimed to determine the antibiotic susceptibility of the bacterial isolates, questions concerning the cause of the infections were not addressed.

In a more recent paper, the antibiotic resistance patterns of *V. cholerae* isolates from local chicken of Bangladesh were determined. The bacteria were frequently found in animal samples, e.g., intestinal fluids, cloacal swabs, egg surfaces [14]. The strains presumably belonged to the toxigenic serogroups causing cholera, and the contaminated chickens were regarded as possible sources of infections for humans. In this study, no disease symptoms of birds were reported.

In 2016 and 2017, the reference laboratory for *Vibrio* hosted at the German Federal Institute for Risk Assessment received two *V. cholerae* non-O1, non-O139 isolates, which were obtained from diseased domestic ducks of a poultry farm in Bavaria. Both incidents caused significant losses of birds and the bacterial isolates were suspected of causing the disease. The unusual source of the isolates prompted this study. To obtain more information on strains from this host organism, two more *V. cholerae* isolates were investigated, which had been recovered earlier in the federal state of Saxony and originated from domestic ducks with serious disease symptoms (in 2011 and 1996).

We studied the four *V. cholerae* non-O1, non-O139 isolates using phenotypic and genotypic methods. Here, we report the results of the comparison of phenotypic traits including antimicrobial resistance patterns and a bioinformatics analysis of their whole genome sequences.

## 2. Materials and Methods

Isolates analyzed in this study are summarized in Table 1. The four investigated *V. cholerae* isolates were recovered in the German federal states Saxony and Bavaria. Isolate 16-VB00145 was identified in a Bavarian poultry farm where ducklings died spontaneously. In two bacteriologically examined ducklings, *V. cholerae* was detected in the liver. Isolate 17-VB00405 was also found in the same farm approximately one year later. In livers of two birds, *V. cholerae* was isolated using a two media cultural approach (Columbia agar with 5% sheep blood and eosin methylene blue agar; Oxoid GmbH, Wesel, Germany). In this case, the incident was recorded by veterinarians and is presented in more details in the results section. The other two isolates were recovered from ducks of Saxonian farms. Isolate CH415 was identified in an incident where ducklings and adult ducks had shown several disease symptoms. Isolate T58 was recovered from a duck that suffered from pneumonia and peritonitis.

**Histology.** Samples from liver, spleen, brain, skeletal muscle and heart were fixed in 10% neutral buffered formaldehyde, paraffin embedded and sectioned with a microtome to obtain 4-μm-thick paraffin sections. H&E stain was performedusing hematoxylin (AppliChem GmbH, Darmstadt, Germany) and eosin (Merck KGaA, Darmstadt, Germany). 

**Species confirmation, PCR typing, and phenotypic characterization.** Species confirmation of *V. cholerae* isolates was carried out by species-specific *sodB* PCR amplification as previously described [15]. The PCR was performed as multiplex PCR and included primers for the detection of the cholera toxin gene *ctxA* [16] and primers for detection of the serogroups O1 and O139 [17,18]. For PCR analysis, genomic DNA (gDNA) was extracted from 1 mL of an overnight culture in lysogeny broth (LB) using the RTP Bacteria DNA Mini Kit according to the manufacturer’s protocol (Stratec Molecular GmbH, Berlin, Germany). Ten nanograms of gDNA served as template DNA for PCR analysis. The primers, annealing temperatures, and amplicon sizes are shown in Table 2.

Matrix-assisted laser desorption/ionization time-of-flight mass spectrometry (MALDI-TOF MS) analysis was conducted for initial species identification using the direct transfer method on a Microflex LT system mass spectrometer (Bruker Daltonik, Bremen, Germany) according to the manufacturer’s recommendation [2]. Additionally, biochemical testing using API20E strips, phenotypic tests for NaCl tolerance, and hemolysis on sheep blood agar were performed.

**Antimicrobial resistance testing**. Antimicrobial susceptibility testing was performed by broth microdilution as previously described [19]. For the determination of the minimal inhibitory concentration the EUVSEC and EUVSEC2 microtiter plates (Trek Diagnostic Systems, East Grinstead, United Kingdom) were used according to the guidelines of the Clinical and Laboratory Institute (CLSI) [20]. The *Escherichia coli* isolate ATCC 25,922 was used as quality control for antimicrobial resistance testing. Interpretive categories of susceptible, intermediate and resistant were assigned according to CLSI clinical breakpoints for *Vibrio* spp. [20]. The agents were chosen according to the harmonized panel of antimicrobials of the European Union EFSA [21].

**Whole genome sequence determination and bioinformatics analysis.** Whole genome sequence (WGS)-based analyses were performed for the four non-O1, non-O139 *V. cholerae* isolates. The preparation of genomic DNA and short-read whole genome sequencing (MiSeq, Illumina, San Diego, CA, USA) was conducted as previously described [22]. SPAdes de novo assemblies of raw reads and genome annotation were performed using the Pathosystems Resource Integration Center (PATRIC) (release 3.5.39) [23] and the automated Prokaryotic Genome Annotation Pipeline (PGAP) of the National Center for Biotechnology Information (NCBI), respectively. The PHAge Search Tool Enhanced Release (PHASTER) was used for prediction of putative prophage sequences under default settings [24]. For the identification of a specific genetic element on the genome sequences of the different isolates, various tools of the Center for Genomic Epidemiology (CGE, Danish Technical University) were applied.

Average nucleotide identity (ANI) was determined with *V. cholerae* O1 El Tor N16961 as a reference genome (AE003852, AE003853) for pairwise comparison with each strain using the online tool ANIFinder (http://enve-omics.ce.gatech.edu/ani/index). The ANI calculations were performed using the default values given on the site (alignment options: minimum length 700 bp, minimum identity 70%, minimum alignments 50; fragment options: window size 1000 bp, step size 200 bp).

Multilocus sequence type (MLST) prediction was conducted using MLST Finder [25] making use of the non-O1, non-O139 *V. cholerae* MLST scheme (https://pubmlst.org/vcholerae/) [26]. Initial plasmid prediction was performed with the PlasmidFinder web tool (release 2.0, https://cge.cbs.dtu.dk/services/PlasmidFinder/) [27]. In addition, genomic contigs showing significantly higher sequence coverages than the rest of the contigs were screened for similarities to known plasmids using the BLASTN algorithm of the NCBI database (https://www.ncbi.nlm.nih.gov).

To determine the phylogenetic relationship of the isolates, a CSI Phylogeny (version 1.4; https://cge.cbs.dtu.dk/services/CSIPhylogeny/)-based single nucleotide polymorphism (SNP) tree was prepared. The tool was used under default settings and the exclusion of heterozygous SNPs. As a reference genome, sequencing data of the *V. cholerae* O1 El Tor strain N16961 (AE003852, AE003853) were used. Nucleotide variations were predicted according to the specifications provided [28]. To find out if closely related genomes of other *V. cholerae* strains were already deposited in public databases, the PATRIC service tool “Similar Genome Finder” (release 3.6.5) was applied. The most similar genomes were tested in pairwise ANI calculations and integrated into the SNP analysis with CSI Phylogeny described above.

For the initial detection of *Vibrio*-specific virulence determinants, the MyDbFinder web-based tool (release 1.1, https://cge.cbs.dtu.dk/services/MyDbFinder/) was used with a manually-adapted composition of determinants selected from the virulence factor database (VFDB; http://www.mgc.ac.cn/VFs/) [29]. The *in silico* prediction of plasmid replicon types and virulence factors and a sequence identity of >50% and >30%, respectively, were used. Furthermore, a minimum coverage of >20% was chosen. For the *in silico* analysis of *V. cholerae*-specific gene variants, the WGS were subjected to the NCBI database (BlastN) and compared to selected reference sequences as previously described [22].

Isolates were further tested for the presence of genes of the Vibrio pathogenicity island 2 (VPI-2) [30]. Isolates were screened for sequences of the Vibrio seventh pandemic islands VSP-1 [31] and VSP-2 [32]. All isolates were further studied for the complete type III secretion system (TTSS) gene cluster [33,34] and for the presence of the MSHA gene cluster. The accession numbers of reference gene clusters are NC_002505.1 (*V. cholerae* N16961; VC1758-VC1809 for VPI-2; VC0175-VC0185 for VSP-1; VC0490-VC0516 for VSP-2; VC0398-VC0411 for MSHA cluster), and DQ124262.1 as well as AATY02000000 (*V. cholerae* AM-19226; AATY02000003.1/AATY02000004.1 for TTSS cluster and flanking regions).

For the presence of specific determinants involved in antimicrobial resistance (AMR) development, ResFinder (release 3.1.0, https://cge.cbs.dtu.dk/services/ResFinder/) was used as specified before. For the prediction of *V. cholerae*-specific integron sequences, the WGS were subjected to NCBI BLASTN searches.

**Accession numbers.** Genome sequences of *V. cholerae* isolates have been deposited in GenBank at the National Center for Biotechnology Information (NCBI) under the accession numbers: PVFA00000000 (CH415), PVFB00000000 (T58), PVER00000000 (16-VB00145), and PVEX00000000 (17-VB00405).

## 3. Results

### 3.1. Case Study in 2017

In the Bavarian poultry farm, ducklings were kept for three weeks in rearing houses (rearing phase) and then fattened for 25–29 days (fattening period) in a mast compartment. In the described incident in the year 2017, 7.8% of ducklings (total 13,515 birds) died during the first three days of the rearing phase. The daily mortality rate decreased on the fourth and fifth day to eight animals (0.06%) and two animals (0.02%), respectively. From then on, daily losses were within acceptable limits (between two to six animals per day). At the end of the third week (day 21), the ducklings were moved to the mast compartment.

Starting at an age of 22 days, however, the losses increased up to 20 animals (0.15%) daily. Therefore, a veterinary examination was carried out on day 25. The animals displayed significant growth differences. The smallest animals could only run poorly and lay on the ground, sometimes they “crawled” through the stable with the help of the wings. A treatment with Suramox 100 mg/g (amoxicillin trihydrate 1000 mg; Virbac veterinary medicine GmbH, Bad Oldesloe, Germany) was immediately initiated with a dosage of 20 mg/kg body weight over four days.

In the first week post treatment, 143 animals died (1% mortality rate). In the following week, the mortality rate decreased to 0.7% (94 animals). All remaining birds were slaughtered shortly afterwards.

**Figure 1:**
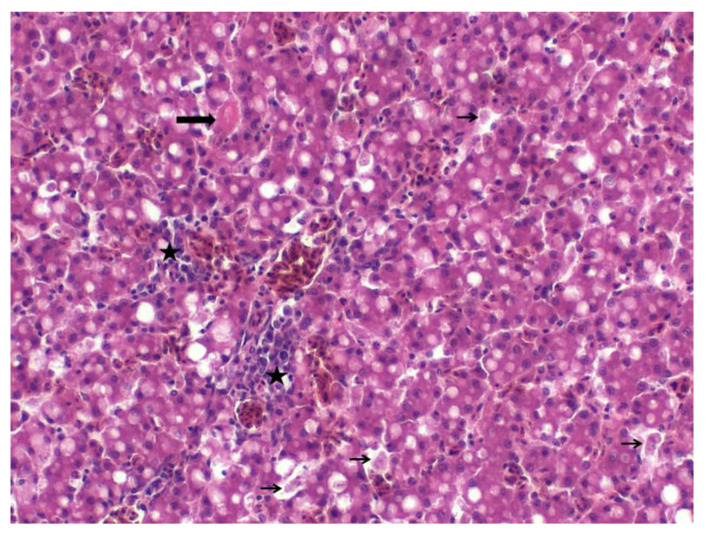
Liver, H&E stain, 200× magnification. Hepatitis with mild degeneration and vacuolation of hepatocytes, heterophilic to mixed inflammatory cell infiltration (stars), small deposits of fibrin (thick arrow), and individual cell necrosis (thin arrows).

Three animals were sacrificed before the onset of antibiotic treatment and presented for necropsy. They were in moderate to poor physical shape. The livers were slightly swollen with rounded edges. Histologically, two livers showed moderate acute degeneration with cytoplasmic vacuolization and single cell necrosis (Figure 1). Additionally, a mild purulent hepatitis with predominantly heterophil granulocytes infiltration was observed. The third liver revealed mild fibrinopurulent hepatitis with perivascular infiltration of heterophil granulocytes and a mild focal precipitation of fibrin. One spleen showed mild fibrin deposits. Other organs, including the brain, heart and skeleton muscle, showed no specific lesions.

A bacteriological examination was undertaken and *V. cholerae* were isolated from two livers and one spleen. In addition, a small number of *Aerococcus viridans* was detectable in one liver. As *A. viridans* is frequently encountered in bacteriological examinations of ducklings, the likely cause of the observed symptoms was attributed to *V. cholerae* by the responsible veterinarians. In the organs of the third bird, no bacteria were found. A pure *V. cholerae* culture (isolate 17-VB00405) was sent to the *Vibrio* reference lab for further investigations.

A systematic investigation of the environment or the water supply system of the farm was not conducted. The water pipes of the farm were disinfected with chlorine after the treatment of the birds with amoxicillin. Different water samples were taken a few days later and were free of *V. cholerae*.

### 3.2. Species Confirmation by PCR, MALDI-TOF MS and Phenotypical Characterization

The results of the multiplex PCR revealed that the four duck isolates belonged to the species *V. cholerae*. In all four isolates, the species PCR (*sodB* gene) yielded the expected PCR product, whereas the multiplex PCR assays were negative for the cholera toxin gene A (*ctxA* gene) and negative for genes specific for serogroups O1 and O139.

All isolates were further analyzed by MALDI-TOF MS using the MALDI Biotyper system with the evaluation criteria developed for species identification. The analyses of the obtained spectra confirmed the species *V. cholerae* (Table 3). Phenotypic characterization showed that the isolates were able to grow in the absence of NaCl and showed hemolytic activity on sheep blood agar. The API20E testing of all strains confirmed *V. cholerae* as probable species (data not shown).

### 3.3. Whole Genome Data

The whole genome sequencing results show that genome sizes of the four sequenced strains vary between 3.9–4.2 Mbp and the average number of putative coding sequences (CDS) is approx. 3.76 × 10^3^. Detailed information of the genomes is given in Table S2.

For each genome, an ANI calculation was performed by comparison with the *V. cholerae* O1 El Tor reference strain N16961. In all cases, the ANI score was >98% (Table 3). A cut-off score of >95% indicates that two strains belong to the same species [35], thus confirming all duck isolates as *V. cholerae*. An MLST analysis of seven housekeeping genes revealed that a considerable difference exists between the strains. Only in one case did two strains share the same allele of one housekeeping gene. This indicates that none of the four duck isolates are genetically closely related. Two new alleles were identified (one *purM* and one *pyrC* allele; see Table S2).

The four genomes were analyzed for their phylogenetic relationship using SNP analysis with the genome of the strain *V. cholerae* O1 El Tor N16961 as a reference. To increase the information on phylogenetic relations, for each of the four strains a search was conducted to detect related *V. cholerae* strains based on genome similarity. For this purpose, the online service “Similar Genome Finder” (PATRIC Server) was applied (accessed 2020-06-14). The closest related genome for each strain was downloaded and included in the tree. ANI calculations, however, indicated that only in the case of strains 17-VB00405 and T58 were more closely related strains identifiable in the data base (data not shown).

**Figure 2:**
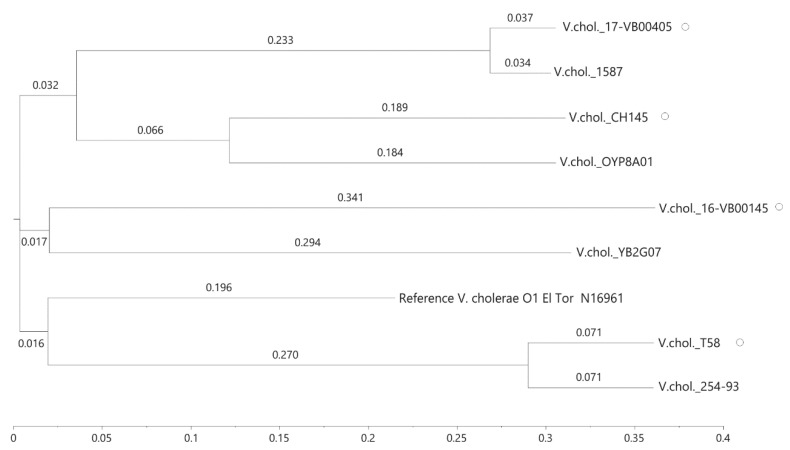
Single nucleotide polymorphism (SNP)-based phylogenetic relationships of *V. cholerae* non-O1, non-O139 isolates. Strains from ducks are marked with a circle. Four similar genomes of *V. cholerae* strains identified by bioinformatics were included in the tree. The SNP tree was conducted using CSI Phylogeny 1.4 under default settings and the exclusion of heterozygous SNPs. Single nucleotide polymorphisms (SNPs) were called by mapping to the *V. cholerae* O1 El Tor N16961 genome as a reference (accession NC_002505.1, NC_002506.1). The scale bar represents the number of nucleotide substitutions per site and numbers indicate branch length. Accession of *V. cholerae* (V.chol) genomes: LBGA01000000 (YB02G07), JMBP00000000 (V.chol 254-93), KQ410623 (V.chol 1587), NMSW00000000 (V.chol OYP8A01).

In total, 3.327.189 positions (corresponding to approx. 82.5% of the reference genome) were used of the investigated genomes and the number of SNPs between the isolates varied between 4888 and 31,855 (Table S3). The differences between the genomes of the duck strains were more than 30,000 SNPs and confirmed that the strains are only distantly related. The SNP-derived tree reproduces these genomic differences (Figure 2). We additionally searched the PubMLST database for *V. cholerae* strains that might be genetically closely related to our isolates; however, no isolate with identical MLST alleles in all seven genes were detected (data not shown).

As *Vibrio* bacteriophages (phages) are common vehicles carrying virulence determinants, the genome sequences were also analyzed *in silico*. The search for prophage sequences revealed that no common prophages are present in the genomes of the four strains. In isolate CH415, a putative intact prophage could be identified using PHASTER. The prophage sequence shows significant similarity to phage K139 (accession NC_003313) (sequence identity >90%) associated with toxigenic *V. cholerae* strains [36]. Another putative prophage sequence of isolate 16-VB00145 revealed only short sequences of similarity to a *Vibrio* phage and was disregarded (data not shown).

### 3.4. Virulence Factors Revealed by WGS

The genome sequences of all strains were screened for virulence genes and the presence of pathogenicity islands. The results of the bioinformatics analysis concerning the presence of virulence genes are shown in Table 4. None of the isolates possesses the major virulence genes of toxigenic O1 and O139 strains that are located on the CTX prophage and the *Vibrio* pathogenicity island-I (VPI-1). The analysis showed that some virulence genes of *Vibrio* pathogenicity island-II (VPI-2) of toxigenic strains are present in strain 16-VB00145, which possess the gene cluster encoding genes for sialic acid metabolism and transport and the *nanH* gene which encodes a neuraminidase. In some non-O1, non-O139 strains, type three secretion systems (TTSS) are present. A TTSS consisting of core genes and flanking 5′ and 3′ regions highly similar to the *V. cholerae* strain AM-19226 T3SS genomic island [33] is found in isolate T58.

In toxigenic strains isolated since 1961 [37], two genomic islands are found that were designated *Vibrio* seventh pandemic islands 1 and 2 (VSP-1, VSP-2). Sequences related to these two islands were mostly absent in the four duck isolates. While no VSP-1 related genes were found at all, genes of VSP-2 were detected in isolate T58. Additionally, a complete VSP-2 island seems to be present in strain 17-VB00405. 

A number of virulence genes can be present in *V. cholerae* non-O1, non-O139 that are also found in the toxigenic strains and are known to contribute to the infection process in a synergistic way [54,55]. The results of a bioinformatics analysis for the presence/absence of a number of these genes are displayed in Table 5. All isolates possess the genes *rtxA* and *rtxC* of the repeat-in-toxin (RTX) cluster. All *rtxA* genes encode a toxin highly similar to the multifunctional autoprocessing repeats-in-toxin toxin (MARTX) of the reference O1 strain N16961 [56]. One of the strains (CH415) may have a functional MSHA pilus, as the *mshA* gene is detected together with a number of secretory genes of the MSHA cluster. The hemolysin genes *hlyA*, *dth* and *tlh* were present in all isolates. The hemolysin gene *trh* associated with the TTSS was found only in strain T58 which harbors a TTSS. All isolates possess genes encoding key proteins involved in quorum sensing (*luxS, cqsA*) and a gene encoding the outer membrane protein OmpU. In all strains, genes for type VI secretion systems (T6SS) are found. The cholix toxin gene (*chxA*) is detected in isolate 17-VB00405.

### 3.5. Antimicrobial Resistance

Antimicrobial resistance testing was performed by broth microdilution. The tests were performed using 19 substances and two combinations (Table S1), whereby the selection of agents follows a standard of the European Union [21].

The isolates were susceptible to most of the tested antimicrobial agents (Table S1). Only one strain (CH415) displayed resistance to trimethoprim and sulfamethoxazole, two agents, which target enzymes of the folic acid metabolism. Furthermore, a high MIC value against nalidixic acid was observed in the same strain. All isolates displayed resistance to colistin. This phenotype is well known in *V. cholerae* and colistin is suggested for selection of this species (cellobiose-polymyxin B-colistin agar) [57].

The bioinformatics analyses using ResFinder revealed the presence of acquired AMR genes in isolate CH415 (Table 5), which harbored the genes *aadA1* (streptomycin/gentamycin resistance), *sul1* (sulphonamide resistance), *catB9* (chloramphenicol resistance), and *dfrA1* (trimethoprim resistance). In isolate 17-VB00405, only a *catB9* gene was present.

The nalidixic acid resistance of strain CH415 may be due to a mutation in the *gyrA* gene leading to an amino acid substitution (aspartic acid to glycine) in position 87.

The AMR genes *sul1, aadA1* and *dfrA1* of strain CH415 were physically linked to a class 1 integron integrase gene *intl1*.

## 4. Discussion

Few studies have been published in which a possible connection between *V. cholerae* and diseases of poultry have been described [10,12]. A disease of laying hens known as “avian vibrionic hepatitis” is a misnomer as the disease is likely to be caused by *Campylobacter* spp. [58].

In this study, isolates that had been recovered from diseased ducks, were characterized as non-O1, non-O139 *V. cholerae* isolates. Two isolates originated from birds of a Bavarian poultry farm, which suffered losses of ducks in 2016 and 2017. An investigation of two deceased ducks in 2016 revealed the presence *V. cholerae* bacteria in the liver of the birds. In 2017, *V. cholerae* was found again in the liver of diseased ducks. Only in the second incident was a limited outbreak investigation carried out. Both incidents point to a possible connection between the isolated bacteria and diseases in birds.

As disease symptoms in waterfowl associated with *V. cholerae* have rarely been reported, two more duck isolates, which had been found earlier in diseased ducks in Saxonian farms, were included in the study. These isolates were recovered from different inner organs (lung, jejunum) and infected animals showed different symptoms. In all cases, an uncertainty remains as to whether the isolates were responsible for the diseases and deaths, as further epidemiological studies were not carried out and an outbreak investigation was only performed once (case study 2017).

WGS was performed and bioinformatics analyses were done to determine possible genetic relationships of the isolates. A search for virulence genes or genomic islands was also conducted, which could indicate factors specific for this animal host. MLST Finder was used to predict sequences of seven housekeeping genes [59]. The MLST data are suited for the characterization of the isolates, as the sequence data are unambiguous and genomes can readily be compared via the internet [60]. Our study disclosed a distinct diversity of the alleles. The two isolates from the Bavarian farm aregenetically distantly related, which is also true for the other two isolates. An SNP analysis of the four genomes with the reference genome of O1 El Tor strain N16961 confirmed this result. Genes of the CTX phage and VPI-1 (toxin coregulated pilus) are missing in the four genomes. In other studies, some non-O1, non-O139 strains were described harboring *ctx, ace, zot,* or *tcpA* genes [16,61].

WGS were performed on environmental *V. cholerae* isolates from other regions of the world. Genomic features of strains from environmental sources from Uganda and Bangladesch [62,63] revealed a genetic relationship to pandemic cholera-causing O1 strains and the possession of the major virulence factors of pandemic strains. In Thailand [64], non-O1, non-O139 strains, which lacked the genes encoded on the CTX phage and for the toxin coregulated pilus, were also studied.

Prophages are frequently involved in the conversion of environmental strains to toxigenic strains. The most notorious case is the acquisition of the cholera toxin gene by integration of the CTX phage into the chromosome of *V. cholerae* O1 and O139 strains [38,65]. However, our analysis did not identify a prophage sequence common to all genomes. In strain CH415, a putative intact temperate phage is present that is related to *Vibrio* phage K139 (sequence identity >90%). K139 is a phage associated with toxigenic *V. cholerae* strains [36,66]. In an animal model, phage K139 was shown to harbor a virulence factor (*glo* gene). However, a *glo* related gene is missing in the prophage sequence of strain CH415.

The search for the presence of pathogenicity island VPI-2 and for two genomic islands associated with toxigenic strains of the seventh pandemic (VSP-1, VSP-2) gave mixed results. VPI-2 genes coding for enzymes of sialic acid metabolism involved in hydrolyzing the intestinal mucus [40] were found in one isolate. A probably complete VSP-2 sequence was present in isolate 17-VB00405 and parts of the island in isolate T58. The role of VSP islands in infection is unclear. It is hypothesized that they increase the fitness advantage of the toxigenic strains [37,67]. In strain T58, a complete TTSS system was identified. TTSS is only present in variants of the VPI-2 found in some non-O1, non-O139 strains and is a known virulence factor in diarrheal diseases caused by these strains [33,34].

In Table 4, the results of a bioinformatics analysis for more virulence genes which are thought to act synergistically in infections are displayed. As in a previous investigation [22], some genes were present in all strains and others were only detected in some strains. Genes of the latter category are, e.g., cholix toxin *chxA* gene (ADP-ribosylating toxin of eukaryotic elongation factor) [51] or the *mshA* gene encoding the major pilin of the type 4 MSHA pilus [42]. The two virulence genes were present in isolate 17-VB00405 and CH415, respectively.

Virulence genes detected in all strains are likely to encode proteins that are probably primarily important for survival and niche adaptation in the natural aquatic environment of *V. cholerae* [68]. These factors comprise, e.g., hemolysin genes (*tlh, hlyA, dth*), genes for quorum sensing (*luxS, cqsA*), the hemagglutination/protease (*hap*), and genes of T6SS mediating antagonistic interactions against many prokaryotic and eukaryotic organisms [47,68]. The occurrence of these virulence factors was observed in all non-O1, non-O139 strains in a previous study [22]. In a study on isolates from Thailand [64], all *V. cholerae* non-O1, non-O139 strains were found to contain *hlya, rtxA* and *toxR* genes, whereas other virulence factors, such as TTSS, MSHA pilus and Elements of the VSP-1, and VSP-2 islands were present only in some strains.

While the bioinformatics analysis did not clearly indicate virulence factors that may be specific for avian hosts, two of the analyzed virulence factors present in all four strains may be of special interest for further research. The OmpU protein is the most abundant outer membrane protein in *V. cholerae*. It is an important virulence factor involved in host–cell interaction and recognition, as well as being critical for the survival in the host body and in harsh environments [69]. The OmpU protein is involved in resistance to antimicrobial peptides in the gut [52]. The primary structures of the four OmpU proteins showed remarkable differences. Whereas the OmpU proteins of CH415 and 17-VB00405 were identical, the proteins of isolates T58 and 16 VB00145 possessed only 77% and 94% identity, respectively. However, C-terminal regions of the protein involved in triggering a bacterial response by the activation of sigma factors were conserved [46]. The significant differences in the primary structures of the proteins may indicate a specific adaptation to different niches or environments.

Another interesting observation was the detection of *rtxA* genes encoding MARTX toxins with a high similarity (>97.7%) to the MARTX of toxigenic O1 El Tor strains. MARTX proteins of *V. cholerae* strains are very large multifunctional proteins (e.g., 4,565 amino acids in O1 strain N16961) with conserved N-terminal and C-terminal regions. The central part of the toxin carries different effector domains [41]. In O1 El Tor strains, the MARTX toxin has three conserved internal domains that are probably involved in the evasion of the host immune defense. In a previous study, we noticed that environmental *V. cholerae* strains frequently possessed MARTX variants differing in the central part of the coding region [22]. These MARTX variants possessed other effector domains and could be active against eukaryotic cells from different organisms (mammals or fish or eels). It was hypothesized that MARTX toxins play a role in adaptation to specific niches in the natural ecosystem [41,68]. Given the variability of MARTX toxins in environmental strains, it is remarkable that all duck isolates harbor the same variant. It was speculated that this variant toxin might be linked to human infections [41].

Acquired AMR genes were found in two isolates (17-VB00405, CH415). The *catB9* gene effective against chloramphenicol was detected and its coding region appeared to be functional. However, in the broth dilution tests, susceptibility to chloramphenicol was observed in the two strains. The *catB9* gene is widely distributed in toxigenic and in non-O1/non-O139 *V. cholerae* strains [64]. In isolate CH415, the AMR genes *sul1, aadA1* and *dfrA1* seem to be linked to a class 1 integron. Class 1 integrons are genetic assembly platforms that take up exogenous open reading frames via site-specific recombinations and are frequently found in *V. cholerae* [70].

## 5. Conclusions

*V. cholerae* non-O1, non-O139 strains are found worldwide in aquatic ecosystems and can cause diseases in aquatic organisms, such as fish and crustaceans. Diseases of waterfowl associated with *V. cholerae* have rarely been reported. For this reason, isolates from diseased birds were of special interest for this study. The analyzed isolates were recovered from different inner organs (liver, lung, jejunum) of domestic ducks and the infections showed different symptoms. Uncertainty remains as to whether the isolates were responsible for the diseases and deaths, as an outbreak analysis was only performed in one case. The great genetic heterogeneity between the isolates does not allow any conclusion about factors contributing to the development in organs of this animal host. Future research is needed and more isolates are necessary to clarify this question. The observation that all four isolates possess MARTX toxins closely related to the MARTX of toxigenic O1 El Tor strains and show differences regarding the highly abundant OmpU protein could indicate a role of these virulence factors in the infection process.

In this context, it should be mentioned that information on the environmental occurrence of *V. cholerae* non-O1, non-O139 in Germany is sketchy. The occurrence of these bacteria in Germany is only documented for coastal waters of the Baltic Sea and some estuaries of the North Sea [71,72]. However, studies from Austria have clearly demonstrated that these bacteria can be present in lakes far away from marine coastal waters [9,73]. As *V. cholerae* non-O1, non-O139 is present in migratory water birds, these birds probably act as vectors for a long-distance transfer of the bacteria. The occurrence of bacteria in inland waters has not been investigated in Germany, so the source of the bird infections currently remains unknown. In the described incident from 2017, the water supply system was suspected to be contaminated (and was disinfected as a precaution). In the context of the ‘One Health’ concept, it is desirable to study the ecology of *V. cholerae* non-O1, non-O139, as it seems likely that the isolated strains from domestic ducks may be also pathogens for humans.

## Figures and Tables

**Table 1:** *Vibrio cholerae* non-O1, non-O139 strains used in this study.

Strain	Year of Isolation	Source	Origin	Information on Disease
17-VB00405	2017	Duck/liver	Bavaria/Germany	Mild fibrinous-purulent hepatitis
16-VB00145	2016	Duckling/liver	Bavaria/Germany	Premature death/stunted growth/lameness
T58	2011	Duck/jejunum	Saxony/Germany	Pneumonia/peritonitis
CH415	1996	Duckling/lung	Saxony/Germany	Dyspnea/polyserositis/pneumonia/inability to stand

**Table 2:** Multiplex PCR for species (*sodB*), cholera toxin gene (*ctxA*) and serogroups O1 and O139 detection.

Gene/Target	Primer	Sequence (5′ to 3′)	Amplicon Size (bp)	T_a_ (°C)	References
*sodB*	VcsodBf	AAGACCTCAACTGGCGGTA	248		[15]
	VcsodBR	GAAGTGTTAGTGATCGCCAGAGT			
*ctxA*	ctxA1	CTCAGACGGGATTTGTTAGGCACG	301		[16]
	ctxA2	TCTATCTCTGTAGCCCCTATTACG		59	
*rfb* O1 cluster	O1F	GTTTCACTGAACAGATGGG	192		[17]
	O1R	GGTCATCTGTAAGTACAAC			
*rfb* O139 cluster	O139F	AGCCTCTTTATTACGGGTGG	449		[18]
	O139R	GTCAAACCCGATCGTAAAGG			

**Table 3:** Species confirmation by Matrix-assisted laser desorption/ionization time-of-flight mass spectrometry (MALDI-TOF MS) and average nucleotide identity (ANI).

Isolate	MALDI-TOF MS		Average Nucleotide Identity **
	Score *	Interpretation	
CH415	2.38	Highly probable identification to species level	98.26%
T58	2.28	Probable identification to species level	98.06%
16-VB00145	2.33	Highly probable identification to species level	98.06%
17-VB00405	2.30	Highly probable identification to species level	98.25%

* Quality of species identification according to Bruker instruction against Bruker main spectra (MSP) libraries. ** Pairwise comparison to genome of strain *V. cholerae* O1 El Tor strain 16961 (accession: NC_002505.1 and NC_002506.1, two-way ANI results): http://enve-omics.ce.gatech.edu/ani/.

**Table 4:** Virulence factors found in duck isolates.

Virulence Factors/Function.	Related Genes	CH415	T58	16-VB00145	17-VB00405	Reference
CTX prophage/cholera toxin	*ctxAB*, *zot*, *ace*, *rstA*, *rstB*, *rstR*	n.d.	n.d.	n.d.	n.d.	[38]
*Vibrio* pathogenicity island 1 (VPI-1)/toxin-coregulated pilus accessory colonization factor	*tcp* cluster, *acf* cluster	n.d.	n.d.	n.d.	n.d.	[39]
*Vibrio* pathogenicity island 2 (VPI-2)/sialic acid (SA) metabolism	VC1776-VC1783 (SA transport, SA catabolism) VC1784 (*nanH*, neuraminidase)	n.d.	n.d.	VC1776-VC1784	n.d.	[40]
Type three secretion system * core region, 5′ and 3′ flanking region	*vsc/vsp* cluster, *vop* effectors, *acfA*, *acfD*, *trh*	n.d.	present	n.d.	n.d.	[33]
*Vibrio* seventh pandemic island 1 (VSP-1)/increased fitness	VC0175-VC0185	n.d.	n.d.	n.d.	n.d.	[40]
*Vibrio* seventh pandemic island 2 (VSP-2)/increased fitness	VC0490-VC0516	n.d.	VC0504-VC0510, VC0516	n.d.	VC0490-VC0516	[37]
Repeats-in-toxin (RTX) toxins/cytotoxin	*rtxA* (similar to VC1451), *rtxB*, *rtxC*, *rtxD*	present	present	present	present	[41]
Mannose-sensitive hemagglutinin pilus (MSHA pilus)/adhesion	*mshA*	present	n.d.	n.d.	n.d.	[42]
Hemolysin genes/cytotoxins	*hlyA*, *tlh*, *dth*	present	present	present	present	[43,44,45]
Outer membrane protein/defense	*ompU*	present	present	present	present	[46]
Type VI secretion system (T6SS) core genes, effectors/interaction	*vipAB*, *vasA-vasK*, *vgrG-2*, VCA0109, VCA0122	present	present	present	present	[47]
Quorum sensing/autoinducer	*luxS*, *cqsA*	present	present	present	present	[48]
Hemagglutination/protease	*hap*	present	present	present	present	[49,50]
Cholix toxin*/ADP-ribosylating toxin	*chxA*	n.d.	n.d.	n.d.	present	[51]
Virulence gene expression/transcriptional activator	*toxR*	present	present	present	present	[52]
Heat-stable enterotoxin *	*stn*	n.d.	n.d.	n.d.	n.d.	[53]

* Factors present in non-O1, non-O139 strains. n.d.—not detected. Gene numbers starting with VC are from genome annotation of O1 El Tor reference strain N16961 (16961 (accession: NC_002505.1and NC_002506.1).

**Table 5:** Phenotypic and genotypic results of antimicrobial resistance of *V. cholerae* non-O1, non-O139 strains.

Isolate	AMP	CHL	CIP	COL	FOX	GEN	NAL *	SMX	TAZCLA	TEMOCI *	TET	TMP	AMR Genes **
**CH415**	4	≤8	0.03	>16	8	2	16	512	≤0.12	4	≤2	>32	*aadA1, catB9, sul1, dfr1, gyrA* (p.D87G) *
**T58**	4	≤8	≤0.015	>16	8	≤0.5	≤4	≤8	≤0.12	2	≤2	0.5	*-*
**16-VB00145**	2	≤8	≤0.015	>16	4	1	≤4	≤8	≤5	2	≤2	0.5	*-*
**17-VB00405**	2	≤8	≤0.015	>16	8	≤0.5	≤4	≤8	≤0.12	2	≤2	0.5	*catB9*

Only selected phenotypes are shown (a complete list is given in Table S1). Gray boxes indicate resistance, MIC concentration in [µg/mL]. Interpretation criteria according to CLSIAbbreviations: AMP ampicillin, CHL chloramphenicol, CIP ciprofloxacin, COL colistin, FOX cefoxitin, GEN gentamicin, NAL nalidixic acid, SMX sulfamethoxazole, TAZCLA ceftazidime/clavulanic acid, TEMOCI temocillin, TET tetracycline, TMP trimethoprim. * no criteria specified by CLSI [20]. ** AMR genes derived from genome sequences are *aadA1* (coding for aminoglycoside nucleotidyltransferase), *catB9* (chloramphenicol acetyltransferase), *sul1* (sulfonamide resistant dihydropteroate synthase), and *dfr1* (dihydrofolate reductase). The *gyrA* gene (nalidixic acid resistance) has a mutation: aspartic acid to glycine in amino acid position 87.

## Supplementary Materials

The following are available online at https://www.mdpi.com/2076-2607/8/8/1104/s1, Table S1: AMR of the duck isolates. Table S2: Results of the whole genome sequence. Table S3: SNP distance matrix of *V. cholerae* non-O1, non-O139 strains.
